# Activities of Daily Living and Determinant Factors among Older Adult Subjects with Lower Body Fracture after Discharge from Hospital: A Prospective Study

**DOI:** 10.3390/ijerph15051002

**Published:** 2018-05-16

**Authors:** Nurul Izzah Ibrahim, Mohd Sharkawi Ahmad, Mohamed S Zulfarina, Sharifah Nurul Aqilah Sayed Mohd Zaris, Isa Naina Mohamed, Norazlina Mohamed, Sabarul Afian Mokhtar, Ahmad Nazrun Shuid

**Affiliations:** 1 Department of Pharmacology, Faculty of Medicine, Universiti Kebangsaan Malaysia Medical Centre, Jalan Yaacob Latif, Bandar Tun Razak, Cheras, 56000 Kuala Lumpur, Malaysia; nurulizzah88@gmail.com (N.I.I.); shark_811@yahoo.com (M.S.A.); szulfarinamohamed@gmail.com (M.S.Z.); sharifahnurulaqilah88@gmail.com (S.N.A.S.M.Z.); isanaina@yahoo.co.uk (I.N.M.); azlina@ppukm.ukm.edu.my (N.M.); 2 Department of Orthopedics and Traumatology, Faculty of Medicine, Universiti Kebangsaan Malaysia Medical Centre, Jalan Yaacob Latif, Bandar Tun Razak, Cheras, 56000 Kuala Lumpur, Malaysia; drsam@ppukm.ukm.edu.my

**Keywords:** older adult, osteoporosis, fracture, disability, ADL, assessment

## Abstract

Fracture is a type of musculoskeletal injury that contributes to an inability to perform daily activities. The objective of this study was to evaluate activities of daily living (ADL) of older adult patients with lower body fracture and to determine factors influencing ADL. Patient’s ADL was assessed at pre-fracture, ward admission and post-discharge phases using the Katz ADL questionnaire. There were 129 subjects at pre-fracture and ward phases and 89 subjects at discharge phase. There were four independent variables; age, gender, type of fracture and ability to walk before fracture. Logistic regression models showed that ‘age’ and ‘ability to walk before fracture’ were the determinant factors of dependent for ‘bathing’, ‘dressing’ and ‘toileting’. The ‘ability to walk before fracture’ was the determinant factor of dependent for ‘transferring’. ‘Age’ and ‘gender’ were the determinant factors of dependent for ‘continence’, while ‘age’ was the determinant factor of dependent for ‘feeding’. The ADL score changes were significant across the phases with a reduction in ADL score in the ward admission phase and partial increment during the post-discharge phase. There were improvements in the health outcomes of subjects aged more than 50 years old after 3 months of being discharged from the hospital. In conclusion, age, being female, having a hip fracture and using a walking aid before fracture were the determinants identified in this study.

## 1. Introduction

Bone fracture is one of the types of musculoskeletal injuries that results in additional injury to soft tissue surrounding the fracture [1,2]. Most bone fractures are the result of injuries from falls or vehicle crashes, but also can be caused by certain diseases, such as tumours and osteoporosis. Osteoporosis is a condition characterized by low bone mineral density (BMD) and micro-architectural bone deterioration, resulting in a loss of bone strength and therefore increased risk of fracture. The risk of osteoporotic fractures, the clinical endpoint of osteoporosis, increases with age. It is associated with deterioration in quality of life, inability to perform daily activities, increased morbidity, mortality and high socio-economic costs [3]. According to the 2016 World Osteoporosis Day report, osteoporotic fractures may affect one in three women and one in five men over the age of 50 [4]. Middle-aged and older men and women with annual height loss of more than 0.5 cm are at increased risk of hip and other fractures, as the bone strength decreases rapidly due to osteoporosis after reaching 50 years of age [5,6].

Fractures can be defined according to the regions of the boney anatomy. Fractures that involves body parts lower than the hip bone are considered as lower limb fractures. This type of fracture accounts for approximately one third of all fractures and may contribute to significant mortality and morbidity [7,8]. Hip fractures have received the most attention compared to other osteoporotic fractures. There are numerous reports on hip fractures contributing to personal burden, especially in terms of limited mobility [9,10]. Typically, hip fractures are associated with loss of ability to walk independently and may require long-term care. Patients may need help carrying out daily activities and thus may be totally dependent on their family members and relatives [11,12,13].

The recovery from an osteoporotic fracture may take time and sustain significant burden in health expenditures: [14] such as hospitalizations, rehabilitative and long-term care, and informal care [15,16]. According to the recent fracture management protocol, patients are discharged from the hospital earlier to minimize costs and reduce risks of nosocomial infections from prolonged hospitalization. This shifts the management of fracture and costs to rehabilitation in the community. A study by Wiktorowicz et al. [17] revealed that the cost of initial hospitalization of hip fracture cases may represent only 27% of the total costs of management, while the costs of managing this patient after discharged from the hospital may account for 69% of the 1-year costs post-fracture. The main cost of rehabilitation was spent on stays at nursing homes and rehabilitation centres, as well as outpatient and home physical therapy sessions [18]. Rehabilitation is the cornerstone in efforts to reduce the rates of ambulatory and functional impairment as well as to improve patient’s activities of daily living (ADL) [19]. While not all patients may experience full recovery of ADL function at hospital discharge, some may experience impaired ADL even months after discharge from the hospital [20]. Thus, it is important to evaluate the ability of fractured patients to perform ADL, as it may provide information regarding the outcomes of osteoporotic fracture patients after their hospital discharge. Apart from that, the relationship between pre-fracture characteristics (age, gender, fracture type and pre-fracture mobility status) and the outcomes of osteoporotic fracture patients after hospital discharge is still unclear and need to be elucidated.

In general, the adverse outcomes in patients who sustained a hip fracture are divided into early complications (occurring within the first 30 to 90 days), and long-term outcomes. Common and potentially preventable early complications of hip fracture may include venous thromboembolism (VTE), delirium, pressure ulcers, cardiovascular events and infections (urinary tract infections, surgical site infections and pneumonia). According to a study by Brown et al. [21], in a single site cohort, one or more events of the early complications occurred in up to 50% of patients who sustained a hip fracture have the inability to walk before fracture. Long-term outcomes that were usually encountered after a hip fracture include functional disability, secondary fractures and mortality. As for functional disability, gait impairment is noticeably common after a hip fracture. Less than half of patient who have had a hip fracture regained their prior level of ambulation at 1 year, and nearly 20% became immobile [22]. Apart from that, patient’s health condition may be worsened by another fracture within two years after their hip fracture, as reported in a study by Colon-Emeric et al. [23]. Eventually, the risk of mortality following hip fracture may be substantially increased.

Although the occurrence of hip fractures has been the subject of many previous studies, the epidemiology of all the combined lower limb fractures that include hip, femur, patella, tibia-fibula, ankle, and metatarsals has not been extensively studied in the Asian population, particularly in Malaysia. In this study, we evaluated the activities of daily living (ADL) of the lower limb fracture patients at three different phases; pre-fracture, ward admission and 3 months post-discharge. Presently, there is very little quantitative information in the literature on the independent variables such as age, gender, types of bone fracture and ability to walk before fracture for lower limb fracture patients. To address these questions, we carried out a population-based study using Katz ADL questionnaire at two hospitals, Hospital Canselor Tuanku Muhriz (HCTM) and Hospital Kuala Lumpur (HKL), which are located in Klang Valley, Malaysia.

## 2. Materials and Methods

### 2.1. Research Design

This study was part of a project called “Malaysia Bone Health and Osteoporosis Study” (MALBONES). This prospective cohort study was conducted in two hospitals around the Klang Valley, Hospital Canselor Tuanku Muhriz (HCTM) and Hospital Kuala Lumpur (HKL), from February 2014 to February 2016. Ethical approval was obtained from Universiti Kebangsaan Malaysia Ethical Committee (UKM1.5.3.5/244/FF-2014-229/Prof. Dr. Ahmad Nazrun Shuid) and the Research Committee Kuala Lumpur Hospital (IRC-IIR/2014/011/156).

### 2.2. Selection and Sample Size of Subjects

Subjects were patients admitted to HCTM and HKL orthopaedic wards. They were selected using selective sampling method based on the inclusion and exclusion criteria. The inclusion criteria were: (1) men and women; (2) aged 50 and above; (3) Malaysian citizens; (4) all types of lower limb fracture; (5) patients admitted to the orthopaedic ward of HCTM and HKL. The exclusion criteria were: (1) patients diagnosed with psychiatric, dementia, Alzheimer’s disease; (2) cancer patients; (3) having pathological fractures other than osteoporosis; (4) patients living outside the Klang Valley; (4) patients with hearing and speech problems.

The sample size of subjects was calculated by using the Green method or formula [24]. The calculation formula based on Green is described as: *N* = 50 + 8 (*m*), where *N* represents “total subjects of study” while *m* represents “independent variable”. In this study, there were four independent variables (age, gender, types of bone fracture and ability to walk before fracture). Therefore, after inputting all the values into the formula, the minimum sample for this study was 82 subjects. However, as this study involved a follow-up visit, drop-out rate must be considered. With the estimated the drop-out rate of 30%, the minimum number of subjects at the start of this study was 107 subjects. In this study, 129 subjects were recruited for the pre-fracture and ward admission phases, and the numbers had dropped to 89 subjects at the post-discharge phase.

### 2.3. Study Protocols

Evaluation of patient’s ADL was carried out at all the phases: pre-fracture, ward admission, and post-discharge phases using validated Katz ADL questionnaire. This questionnaire was chosen because of its widespread use in the evaluation of ADL status, particularly for older adult subjects [25,26,27]. Data collection for the pre-fracture and ward admission phases were performed when the subjects were in the ward. While, for the post-discharge phase, subjects were assessed during their follow-up visits at their respective orthopaedic clinics three months after discharge from the hospital. All data were based on self-report by interviewing the subject, while the questionnaire was filled out by the researcher.

The Katz ADL questionnaire consisted of a score that reflect whether the subject is ‘dependent’ or ‘independent’ on the six items related to self-care (bathing, dressing, toileting, transferring, continence and feeding). “Independent” is defined as the ability of the subject to perform an unattended activity, direction or assistance and is based on the current status. ‘Dependent’ is defined as a subject that requires assistance, supervision and direction for the activities. Each of the daily activity in this questionnaire is also defined in detail with the assessment of whether it is ‘dependent’ or ‘independent’ for each subject.

‘Bathing’ in ADL status is defined as ‘dependent’ for subjects that need help to cleanse more than one body part during bathing, need help to get out and into the bathroom. Meanwhile, ‘independent’ in terms of ‘bathing’ is defined as being able to shower by themselves or need help to cleanse only one body part. For ‘dressing’ activities, subjects are assumed to be ‘dependent’ if they are unable to dress themselves fully. Subjects are categorized as ‘independent’ in ‘dressing’ if they are able to pick up garments in clothes storage, and wear clothes on their own. Apart from that, if subjects are not able to tie the shoe strap, they are still categorized as ‘dependent’ in ‘dressing’.

For the ‘toileting’ activity, subjects are considered ‘dependent’ if using catheters or need help (supervision, instruction and personal assistance) with toileting. Subjects who are able to go to the toilet, and clean the urinary and digestive organs are categorized as ‘independent’. The ‘transferring’ activity is defined as ‘dependent’ if subjects require assistance (supervision, instruction and personal assistance) in moving from the bed and/or chair and also unable to perform one or more movements. Subject are defined as ‘independent’ if able to move from bed and/or chair on their own. The use of mechanical aids is permissible.

The ‘continence’ activity is defined as ‘dependent’ if subjects have problems in controlling or carrying out urination or defecation partially or fully. Subjects that depend on auxiliaries for stool discharge such as enema, catheters or frequent use of urinal or stool containers are assumed to be ‘dependent’ on ‘continence’ activity. Subjects that are capable of controlling urination and defecation themselves are defined as ‘independent’ in ‘continence’ activity. The ‘feeding’ activity is defined as ‘dependent’ if subjects require assistance to feed i.e., needing personal help to put food into the mouth or using tubes and not taking food on their own. Subjects capable of self-feeding without assistance are defined as ‘independent’ for ‘feeding’ activities. Food preparation is not included in this assessment. Each subject was assessed for every daily life score by giving ‘0′ for the ‘dependent’ subject and ‘1′ point for the ‘independent’ subject. Scores are added to get the score for six ADL items. The total for this assessment score ranges from 0 to 2 (dependent), 3 to 4 (semi-dependent) and 5 to 6 (independent) [28,29,30,31,32].

### 2.4. Statistical Analysis

Descriptive analysis was conducted to determine the frequency and the percentage of the subject for each ADL item. Subjects were analyzed either ‘dependent’ or ‘independent’ for each ADL item for all the three phases (pre-fracture, ward admission and post-discharge phases). Logistic regression models were performed to define the determinant factor of ‘dependent’ after three months post-discharge for each ADL items.

Apart from that, receiver operating characteristic (ROC) test was performed to determine the stability of the logistic regression models. The value of area under the curve (AUC) was used as a reference to determine the strength of a model. The value of area under the curve (AUC) is characterized according to the interpretation values: (i) Excellent (0.91–1.00); (ii) Good (0.81–0.90); (iii) Fair (0.71–0.80); (iv) Weak (0.61–0.70); (v) Unacceptable (0.50–0.60) [33]. Descriptive tests were conducted to determine minimum and maximum scores and mean scores of ADL for the three phases. Besides that, Friedman test was conducted to assess whether there was a significant increase or decrease in scores of ADL for the three phases. The Mann Whitney U test also was conducted to determine whether there was a significant difference between independent variables with ADL scores after three months of discharge from the hospital.

## 3. Results

A total of 129 subjects were enrolled for the pre-fracture and ward admission phases, and there were 89 subjects left at 3 months post discharge phase. The demographic data of the patients is tabulated in Table 1. Of the 40 subjects who dropped out, 14 had died, 15 had withdrawn from the study and 11 were uncontactable (Table 2).

### 3.1. ADL: Frequency and Percentage of Independent or Dependent

At the pre-fracture phase, the majority of patients were “independent” for all ADL assessed in this study (bathing, dressing, transferring, toileting, continence and feeding). A total of 128 subjects (99.2%) were ‘independent’ in bathing, dressing, transferring and toileting while only one subject (0.8%) was categorized as ‘dependent’. In terms of continence, 113 subjects (87.6%) were ‘independent’ and 16 subjects (12.4%) were ‘dependent’. Feeding activity of ADL showed that all 129 subjects (100.0%) were ‘independent’.

In the ward admission phase, feeding was the highest ADL ‘independent’ activity with 110 of the subjects (85.3%) categorized as ‘independent’ and only 19 (14.7%) subjects were categorized as ‘dependent’. In terms of bathing activity, only 2 subjects (1.6%) were ‘independent’ compared to 127 subjects (98.4%) who were ‘dependent’ of this ADL activity. Dressing activity showed that 14 (10.9%) subjects were ‘independent’, while 115 subjects (89.1%) subject were ‘dependent’. A total of 12 (9.3%) subjects were ‘independent’ in transferring activity and 117 (90.7%) subjects were considered as ‘dependent’. Toileting and continence activities showed that 3 (2.3%) and 22 (17.1%) subjects were ‘independent’ while 126 (97.7%) and 107 (82.9%) subjects were ‘dependent’ respectively.

The post-discharge phase showed more independency compared to the ward admission phase for all ADL activities. Feeding activity showed the highest improvement with 84 subjects (94.4%) ‘independent’ and only five subjects (5.6%) ‘dependent’. In terms of continence activities, 66 subjects (74.2%) were ‘independent’ and 23 subjects (25.8%) were ‘dependent’. Transferring activity showed that 67 subjects (75.3%) were ‘independent’ while 22 subjects (24.7%) were ‘dependent’. A total of 57 subjects (64.0%) were ‘independent’ in dressing activity and 32 subjects (36.0%) were ‘dependent’. Both bathing and toileting ADL activities showed the least improvement in independency, with only 55 subjects (61.8%) declared ‘independent’, while, 34 subjects (38.2%) were still in ‘dependent’ category. The frequency and percentage of subjects according to ADL scales (independent/dependent) in all three phases were listed in Table 3.

### 3.2. Determinant Factors Dependent for ADL at Post-Discharge Phase

Six logistic regression models were developed to assess the relationship between the six ADL scales and independent variables. The post-discharge phase (three months after discharged from the hospital) was used to be tested against four independent variables: ‘age’, ‘gender’, ‘fracture type’ and ‘ability to walk before fracture (use or did not use walking aids)’.

The first regression model developed was for the ADL bathing activity and the independent variables. According to the model, only two variables showed significant value; ‘age’ (*p* = 0.036) and ‘ability to walk before fracture’ (*p* = 0.005).While, the other two variables; ‘gender’ and ‘fracture type’ did not show any significant changes. The strong determinant factor for bathing activity was the use of walking aid before the fracture with an odd ratio (OR) of 5.87. This means that, by controlling other factors in the model, subjects that used walking aids before fracture have 5.87 times higher probability of becoming ‘dependent’ on bathing activity than subjects that did not use the walking aids before fracture. ‘Age’ factor showed an OR of 1.08, meaning that by controlling other factors in the model, increased age by one year may increase the probability of becoming ‘dependent’ on bathing activity by 1.08 times.

The second regression model was developed for the determinant factor of dressing activity. Two factors showed significant value; age (*p* = 0.001) and ability to walk before fracture (*p* = 0.001). Both gender and fracture type factors did not show any significant value, with *p* value of 0.67 and 0.35, respectively. Both age and the use of walking aids before fracture factors showed an OR of 11.23, suggesting that these two factors may increase the probability of becoming dependent for dressing by 11.23 times. Based on the regression model of determinant factors for transferring activity, only the ability to walk before fracture showed significant value of *p* = 0.029, while other factors did not show any significant value. The OR for subjects that used walking aids before the fracture was 3.68, suggesting that the probability of being dependent for transferring activity was 3.68 times higher in subjects that had used walking aids.

As for toileting activity, it was shown that age and the ability to walk before fracture had significant value of *p* = 0.043 and *p* = 0.011, respectively. The OR for age factor was 1.07, while the OR for subjects that used walking aids before the fracture was 4.62. The OR suggested that a one-year increase in subject’s age increased the probability of dependency for toileting activity by 1.07 times, while subjects that used walking aids before the fracture have the probability of being dependent on toileting activity by 4.62 times higher than subjects that did not use walking aids before the fracture.

The fifth regression model was generated for continence activity. Age (*p* = 0.013) and gender (*p* = 0.043) showed significant values. The OR for age was 1.12, suggesting that a one-year increase in age increased the probability of being dependent for continence activity by 1.12 times. The OR of 4.35 for female subjects showed that the probability of being dependent for continence activity was 4.35 times compared to male subjects.

The final regression model was for feeding activity. Only the age factor (*p* = 0.019) was significant. The age OR was 1.40, suggesting that a one year increment increased the probability of being dependent for feeding by 1.40 times. Details of the logistic regression were tabulated in Table 4.

**Figure 1:**
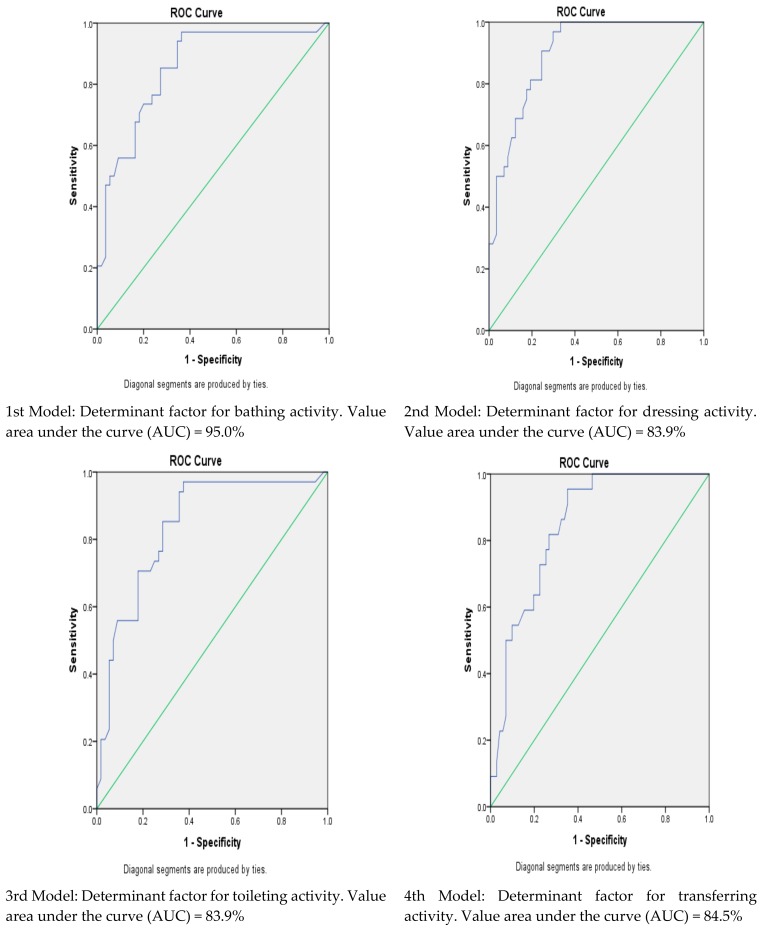

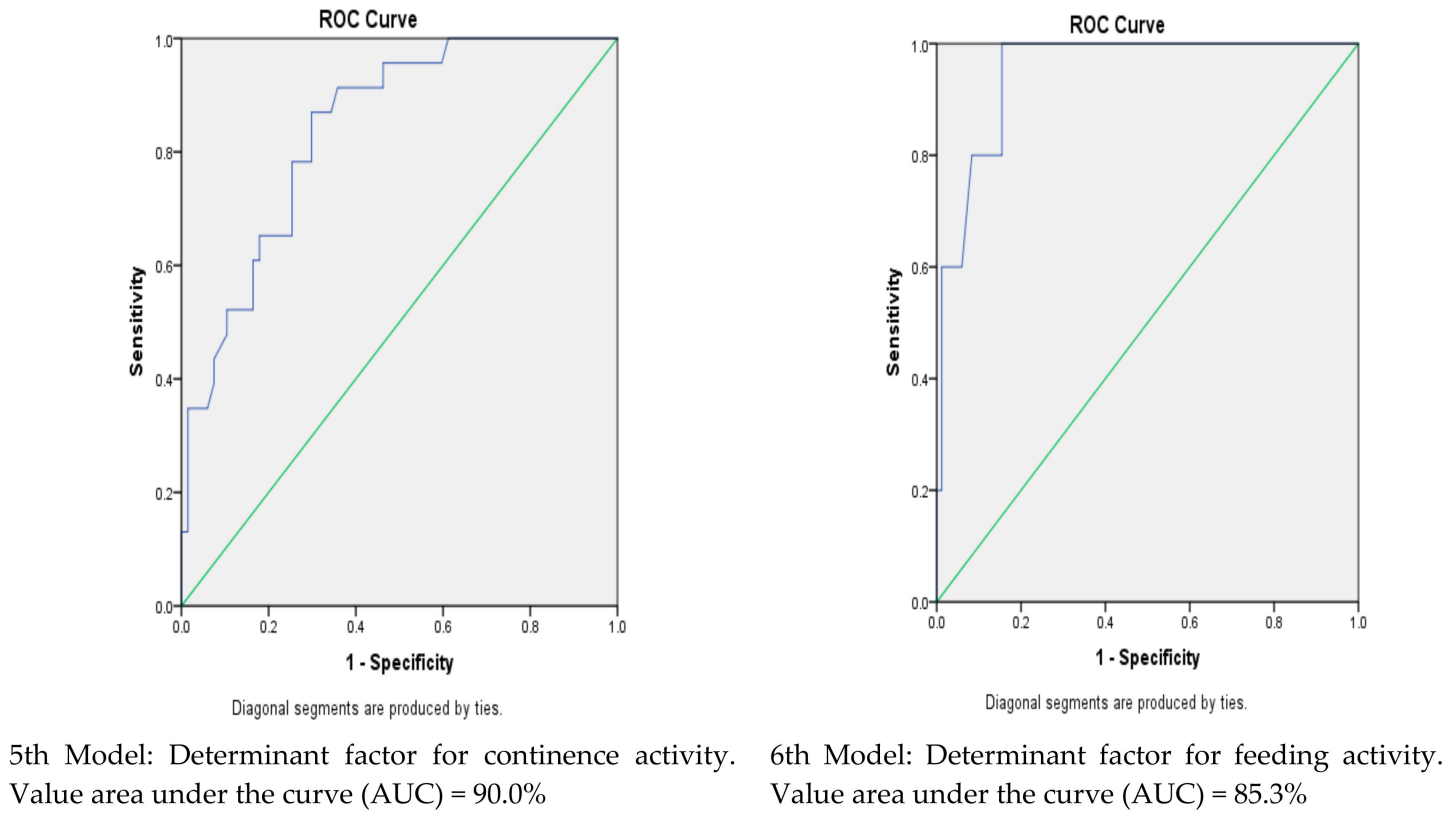
ROC curve with AUC values for all the six regression models.

To determine the strength of the regression models, the receiver operating characteristic (ROC) analysis test was performed. For the first model, the determinant factor for bathing activity, showed the area under the curve (AUC) of 95%. Both second and third models, the determinant factor for dressing and transferring respectively, showed the AUC of 83.9%. The AUC for the fourth model (the determinant factor for toilet activity) and fifth model (determinant factor for continence activity) showed the AUC values of 84.5% and 90.0% respectively. The final and sixth model (determinant factor for feeding activity) showed the AUC value of 85.3%. Figure 1 showed ROC curve with AUC values for all of the six regression models. All the models have AUC values in the good category and acceptable.

### 3.3. Daily Life Activity Scores (ADLs) across All Three Phases of Study

From the data analysis, the mean daily activity score (ADL) in the pre-fracture phase was 5.85 which can be categorized as ‘independent’. In the ward admission phase, the mean score was 1.28, which was categorized as ‘dependent’. For the post-discharge phase, the ADL score was categorized as ‘partial dependent’ with a mean score of 4.33.

The Friedman test was conducted to determine if there were significant changes for ADL scores across all three phases. There was a significant change (*p* = 0.000) on ADL scores across all three phases in this study. The results showed that there was a decrease in ADL scores from the pre-fracture phase with median score of 6 and inter quartile range (IQR) of 0 to the ward admission phase with median score of 1 and IQR of 0. Then, there was an increase in ADL scores from the ward admission phase to the post discharge phase with median score of 6 and IQR of 4. Table 5 shows minimum scores, maximum scores and mean scores for all three phases and Table 6 shows changes in ADL scores across all three phases.

### 3.4. ADL Score and Independent Variables (Age, Gender, Type of Fracture and Ability to Walk before Fracture)

The analysis of the Mann-Whitney U test revealed that all independent variables (age, gender, fracture type, ability to walk before fracture) showed significant difference with the ADL scores in post-discharge phase. There was a significant difference (*p* = 0.000) between subjects in the middle age and older adult categories. Subjects in the middle age category (50–64) had a higher score with a median IQR of 6 (0) compared to the older adult age category (≥65) with a median IQR score of 3 (5). There was also a significant difference (*p* = 0.021) between the gender in which median IQR of the male and female subjects were 6 (one) and 6 (five), respectively.

The independent variable of fracture type showed significant values (*p* = 0.000) with the hip fracture type with median IQR of 3 (five) and non-hip fracture with median (IQR) 6 (zero). The hip-fracture type had lower median IQR score. Subjects using walking aid before fracture were significantly different in ADL scores (*p* = 0.000) compared to subjects not using walking aid before fracture. Subjects walking aid before fracture had lower median IQR of 2 (three) compared to subjects not using walking aid before fracture, with median IQR of 6 (two). Table 7 showed ADL scores and independent variables (age, gender, fracture type and ability to walk before fracture).

## 4. Discussion

### 4.1. The scores of Daily Life Activity (ADL) Scale

The Katz ADL questionnaire was the main tool used to measure and assess functional performance in patients [31,34]. It was established to evaluate functional outcomes in older adult by rating the subjects as dependent or independent of the six scales of basic activities; bathing, dressing, toileting, transferring, continence and feeding [35]. It is sensitive to detect declining health status but it has limited ability to measure small increments of change during rehabilitation of older adults. However, it is very useful in creating a common language to evaluate the functional outcomes in older adult for an overall care planning and discharge planning of all practitioners and caregivers [36].

There are various factors that lead to difficulties in doing all the basic activities. Most patients diagnosed with osteoporosis, arthritis and stroke have higher potential in ADL difficulty. Older adult, especially those aged 85 years and above, have a higher problem in performing ADL than younger ones [37]. This study focuses on ADL-related assessments among subjects aged 50 years and above and the evaluations are based on the three main phases after a fracture i.e., pre-fracture phase, ward admission phase and the post-discharge phase (3 months after hospital discharge). In this study, the four variables (age, gender, fracture type and ability to walk before fracture) were chosen in accordance with several previous studies that these variables contribute much influence on ADL [20,25,26,27]. Thus, this study was performed focusing on the Asian population, particularly in Malaysia.

In this study, the pre-fracture phase showed that most of the subjects (99.2%) were ‘independent’ in bathing, dressing, transferring and toileting. This result was in line with the results of Puteh et al., who studied elderly subjects in four districts in Selangor, Malaysia. In the study, it was shown that among elder subjects (60 years and above), there was a higher percentage (93.1%) of independent subjects on basic ADL activities as compared to dependent subjects (30.7%). However, the comparison between these two studies was limited by the difference in questionnaires used to assess the elder subjects. Puteh et al. used the Modified Barthel Index (MBI) to assess self-reported ADL dependency. The aspects evaluated in the questionnaire and the scoring of ADL are quite different, and these make it difficult for a scrutiny comparison regarding the daily activities [38].

In our study, feeding activity showed the highest percentage of ‘independency’ compared to other ADLs. This result corresponded to a study conducted among older adult over 60 years old in Beijing, China in 1999 [39]. In the study, feeding activity for all the subjects (100%) was in the ‘independent’ category. However, direct comparison with our study cannot be carried out as the Beijing study used a different ADL evaluation questionnaire with “14 self-reported questions” [39]. Apart from that, the results of our study were consistent with the findings of the study conducted in Brazil among the older adult aged 60 years and above. The study in Brazil was conducted in urban areas and used the same questionnaire, Katz ADL. Therefore, a more detailed comparison of each activity can be made more clearly and accurately. The findings showed that most of the subjects in the older adult were categorized as “independent” for basic activities such as bathing, dressing, transferring, toileting, and feeding. These coincided with our findings. Moreover, the study showed that continence activity had the highest percentage of dependence compared to other ADLs, with 21.3% subjects categorized as dependent (partial/full assistance) [25]. This was also in line with our study, in which continence also showed the highest percentage (12.4%) of dependent subject. The high “dependency” in continence activity can be defined as dual incontinence of urine and stool, which is the most extreme manifestation of pelvic floor dysfunction. The dual incontinence is associated with a greater negative effect on quality of life and is more prevalent among older adults [40]. Thus, this might be the reason of the high “dependency” in continence activity during pre-fracture phase.

Although the decline in ADL activity was usually closely related to age factors [20,25], several previous studies including ours, showed that prior to fracture, most older adult could still be categorized as ‘independent’ for most ADL activities [20,41]. For instance, in our study, majority of the subjects were independent in all ADL activities before getting the fractures. Thus, age is not the major cause of difficulties in doing basic day-to-day activities, but, trauma such as fracture occurrence can contribute to dependency in doing ADL activities [41,42]. This could be clearly seen in our study, where after the event of a fracture (ward admission phase), the majority of the subjects had very significant deteriorations in every aspect of ADL activities. The five ADL activities such as bathing, dressing, transferring, toileting and continence showed high “dependency” percentage of more than 80% during the ward admission phase. Only feeding activity maintained a high ‘independent’ subject of 85.3% in the ward admission phase. This finding was supported by a study by Kelsey & Samelson (2009), which concluded that fractures in older adult occur commonly at hip or proximal humerus, in which feeding activity is not directly affected [43].

The outcomes in ADL function after a fracture were reported to be inconsistent among several studies. In a systematic review study conducted by Sharkawi et al., the outcomes of the ADL function using the Katz ADL questionnaire were found to be different. Of the five studies included in the systematic review, three studies showed increased ‘dependency’ levels on the patient’s ADL after a hip fracture. While, two other studies showed reduced ‘dependency’ levels with less than half of the fracture patients failed to regain their pre-fracture ADL level after one year of a hip fracture [26]. The different outcomes for these studies may be contributed by the different study duration and the methods of follow up. The duration of follow up visit varied; 6 months to 4 years [44], 1 year [30,45,46] and 8 years [47]. With regards to methods of follow up, other than home visits, the patients were also contacted via email or telephone, which might affect the responses.

In our study, the ADL activities, except for the feeding activity, showed the percentage of independent between about 60 to 74%. This was parallel to a study conducted by Alarcon et al. where patients who have had a hip fracture were followed-up for 24 months to measure their improvements in activities of daily living. In this study, activities such as climbing stairs, chair/bed transfers, ambulation, dressing, bathing and use of toilet showed the percentage of independent between 67.5 to 76%. However, this study had used a different questionnaire, which was “Barthel Index” questionnaire. Apart from that, the duration of follow-up was also different, in which the subjects were followed up for 2 years [48].

In our study, both the bathing and toileting activities showed the highest ‘dependency’ compared to other ADLs during the ward admission and post-discharge phases. Meanwhile, feeding showed the lowest ‘dependency’ among the subjects in all three phases. Bathing and toileting activities were the ADL activities that largely depended on the lower limb functions. This may be the reason why patients with lower limb fracture became dependent on assistance [49]. Magaziner et al. reported that for patients with lower limb fractures, the recuperation period were 11 months for lower extremity physical and instrumental ADLs. This showed that the recovery period for lower limb fractures were quite long, and these patients were in serious need of help to do ADL with lower probability of recovery [50]. Alarcon et al. had followed-up patients who had a hip fracture for 2 years to assess recovery to their previous pre-fracture level. They classified the activities of daily living into two categories; (i) lower probability of recovery (climbing stairs, chair/bed transfers, ambulation, dressing, bathing and use of toilet), (ii) higher probability of recovery (grooming, feeding and bladder and bowel control). Thus, this study was in agreement with our study, which showed bathing and toileting having the lower probability of recovery. Apart from that, both the studies reported that feeding had the lowest ‘dependency’ among the subjects [48]. The functional decline of older patients is closely related to reduced physical activity as a result of pain and afraid of re-fracture. According to Riemen and Hutchison, encouraging the fracture patients to remain physically active and participate in self-care can reduce the declined functions in ADL [51]. Apart from that, the management of fracture patients should include intervention programmes to prevent fracture and to improve rehabilitation and treatment for older patients with lower limb fracture.

### 4.2. Determinant Factors for Daily Life Activities at Post-Discharge Phase 

Several studies have revealed that the prevalence of trauma and recovery from post-traumatic injuries were affected by age, gender, and living alone [52,53]. Other identified determinant factors for ADLs after traumatic bone fracture includes existing health/illness, residence, rehabilitation protocol and type of bone fracture suffered. Factors affecting or impacting the ADLs of traumatized older adult with bone fractures can be influenced by different cultures and health-related systems for each country. Some countries may lack comprehensive programme for prevention, treatment, and long-term rehabilitation for older adult trauma patients. Thus, determinant factors for ADL after trauma should be fully understood to ensure proper planning and strategies in the rehabilitation programme [20].

Most of the existing studies explained the determinant factors for ADL on the overall scores of ADL scales. However, detailed studies on determinant factors for each ADL scale is limited and rarely done. To the best of our knowledge, this is the first detailed study on determinant factors for all the ADL activities (bathing, dressing, transferring, toileting, continence, and feeding) among patients with lower limb fracture, especially in Malaysia. The results of this study can be used as a reference for future studies in improving the effectiveness of rehabilitation and recovery of bone fracture patients. Six logistic regression models were tested to identify the determinant factors for each ADL scale at the post-discharged phase in this study.

In our study, age was the determinant factor for ADL activities such as bathing, dressing, toileting, continence and feeding. This finding was consistent with two previous studies that showed increasing dependence with age [20,54]. This may be due to the fact that elders usually have many health problems such as musculoskeletal disorders, visual and hearing impairment which deteriorated with age [55]. There is also a possibility that elder patients may limit their activities after trauma in fear of re-injury, and this contribute to age being the determinant factor for ADL activities. According to a study by Povoroznyuk et al., the reparative processes for fracture in older adult were slower compared to younger population [56]. This might be due to the decrease in the activity of genes responsible for cell regeneration with age [57].

Gender was the determinant factor for continence activity, in which female subjects showed the odds ratio of 4.35 after other factors had been controlled in the model. The odds ratio indicated that female subject had 4.35 times higher probability of being dependent for continence activity compared to male subject. Difficulties in continence are closely related to pelvic floor disorders, and this is more associated to women. According to Matthews et al., they identified a strong association between multiparity and dual incontinence. It is possible that cumulative effects of anatomic and neuropathic problems in older women are more likely to result in the pelvic dysfunction and contribute to dual continence problem [40]. Apart from that, according to Nitti, the prevalence of incontinence in women is relatively low early in life, peaking around the time of menopause and then rising steadily between the ages of 60 to 80 years. The prevalence of incontinence is much lower in men than women [58]. Stenzelius et al. found that the prevalence of self-reported symptoms of urinary, faecal, and double incontinence in subjects of 75 years and older showed significant changes (*p* < 0.001) in urinary incontinence among women, compared to men [59]. Thus, these evidences may support the findings of our study on continence activity.

According to all logistic regression models performed, type of fracture was not the determinant factor for any impaired function with ADL activities. However, hip fracture is considered the most serious fractures for older adult, resulting in impaired function, and increased morbidity and mortality. Functional ability, specifically related to activities of daily living, is limited in hip fractures, and according to published data, elder patients with hip fracture did not reach their pre-fracture levels of functioning one year post-fracture in 29 to 50% of cases [60,61]. Apart from that, hip fractures are associated with a marked decline in physical functioning at 2 years, independent of the effects of increasing age, pre-existing medical conditions and disabilities [62].

In the present study, use of walking aid before fracture was a determinant factor for daily life activities such as bathing, dressing, transferring and toileting. However, use of walking aid before the fracture was not a determinant factor for continence and feeding activities after controlling other factors in the model. From the finding, it can be seen that subjects who used walking aid before a fracture have higher difficulties in performing ADL activities. These were closely related to activities involving movements, mobility and activities that use lower body parts such as bathing, transferring, dressing and toileting. The findings of this study was in line with a study conducted by Mariconda et al. that evaluated the status of ADL among hip bone fractures patients after one year using the same questionnaire, Katz ADL. The study demonstrated that limited ability to walk before fracture was identified as the determinant factor for patient outcomes after a bone fracture [63].

All the logistic regression models performed in determining ADL’s dependent factors have values that could be categorized into a fair category, which meant that the models were strong and acceptable. The average score of the area under the curve (AUC) for all the models were greater than 80% [33].

### 4.3. Daily Life Activity Scores (ADLs) Across All Three Phases of Study

ADL scores and classification using the Katz ADL questionnaire are typically used to detect problems in performing activities of daily living and to plan care accordingly. In this present study, Katz ADL scores were categorized into three main categories of score 0–2 (dependent), score 3–4 (partial dependent) and score 5–6 (independent) [36].

It was shown that in the pre-fracture phase, subjects were categorized in the ‘independent’ group with a mean score of 5.85. The results of this study were in parallel with that of the study conducted among 65-year-olds and above in Taiwan. However, the study in Taiwan used a different questionnaire, but with the same evaluation of ADL activities (bathing, toileting, transferring, dressing, continence and feeding). It showed that the prevalence of difficulties in performing ADL was low, ranging from 7% to 9.3% [64]. Besides that, our study was also similar to the Hong Kong study conducted among the older adult Chinese aged 65 years and above. The Hong Kong study, which also used the Katz ADL questionnaire, showed only 1.4% of male subjects and 1.7% of female subjects experienced difficulties in performing ADL [27]. These meant that most older adult who do not have bone fractures were categorized as independent in ADL. In addition, a study conducted among older adult aged 65 years and above without fractures at four outpatient centres in Penang, Malaysia have also shown similar results, with the mean value at 5.30 and categorized as ‘independent’ [28].

Most previous studies did not evaluate ADL scores of patients while they were in the ward. However, this present study assessed the ADL scores of patients in the ward (ward admission phase). During this phase, the mean score of ADL was very low; 1.28 and this was categorized as ‘dependent’. The subjects may still be traumatized and still receiving treatment. According to Inagawa et al., (2013), lower limb fractures in older adult have more intense effect on ADL scores contributing to reduction in their ability to perform basic activities [65].

The mean score of ADL for 3 months post-discharge was 4.33 and this was categorized as ‘partial dependent’. This showed that the subjects failed to regain their pre-fracture level on ADL activities at three months after discharge. Most functions in activities of daily living could be achieved by four to six months after the fracture [66]. According to Magaziner et al., the recuperation times were specific to the area of function, ranging from four months for upper extremity function to almost a year for lower extremity function [50]. Thus, patients with lower limb fracture need longer recovery time to gain their pre-fracture ADL abilities.

Following the Friedman test, there was a significant reduction of ADL scores from pre-fracture phase to ward admission phase. This signified the limitation on ADL activities of the patient after fracture incident. From then on, there was a significant increment of ADL scores from ward admission phase to 3 months post-discharge phase. This meant that fracture healing process were on-going with improvement in the ADL activities.

### 4.4. ADL Score and Independent Variables (Age, Gender, Type of Fracture and Ability to Walk before Fracture)

There are multiple factors which can influence patient’s outcomes after a hip fracture: age, pre-fracture functioning and health status, fracture type, associated pain, anaemia, dementia, muscle strength, and early mobility level [67,68]. Thus, the present study found that all independent variables tested (age, gender, fracture type, ability to walk before fracture) were significantly different from the ADL scores in post-discharge phase.

Al-Ani et al. [69] stated that the epidemiology of fracture in the older adult has been extensively investigated, but there were few prospective studies conducted among middle-aged patients. In the present study, the ADL scores of middle-aged patients were compared to older adult subjects. It was shown that middle-aged subjects had higher ADL score compared to older adult subjects. The ability to perform ADL among fractured patients were closely related to age. This was supported by Alegre-Lopez et al. who reported that age more than 80 years was one of the independent predictor of limited functional ability after a hip fracture incident [70]. However, Curry et al. reported that it was still possible for older women who experienced hip fracture to achieve an ‘independent’ functional level of ADL. This was not in agreement with our study although the same Katz ADL was used. However, the sample size of that study was only 23 Caucasian hip fractured women [44], which were greatly different to our sample size of 129 female and male subjects. This may contribute to the inconsistent result.

Patients who sustained a hip fracture, regardless of gender or age were seen to receive impact in ADL [71]. Previous studies on gender and ADL recovery have produced inconsistent results [72,73,74], suggesting for further research to clarify this issue. A systematic study was conducted to examine gender differences in hip fracture outcomes but was unable to make any concrete conclusions pertaining to gender difference and the outcomes function after hip fracture [75]. In the present study, significant differences in gender were found, with female at higher risk of being ‘dependent’ in ADL activities. A cohort study by Alegre-Lopez et al. on patients who have had a hip fracture showed that female gender was one of the independent predictors of limited functional ability other than age, poor mental status and pre-fracture functional disability [70]. The study had used a different questionnaire which was “MEDOS” and the duration of the follow-up visit was one year after a hip fracture. A different study conducted among older adult who suffered from falling injuries such as hip fractures, other bone fractures and other injuries showed that female had lower ADL recovery rates compared to men after one year of injury [76]. This time, Groningen Activity Restriction Scale (GARS) was used as a tool for evaluating ADL. In addition, the number of male subjects (31 subjects) was much lower than that of female subjects (140 subjects) and this unequal ratio may indirectly influence the result of the study. In a study by Garcia et al. which examined functional outcomes among senior citizens with hip fracture in Brazil using the Katz ADL questionnaire showed that male had decreased functional status after one-year fracture. However, the sample size of the study was very low, 29 male and five female subjects. The sample size and unequal ratio of male to female may have influenced the study result [30].

Based on our knowledge, studies that compare the types of fractures (hip and non-hip fracture) to the scores of ADL scale are very limited. This study found a significant difference in the ADL score after three months of discharge from the hospital between subjects with hip fracture and non-hip fracture. Subjects with hip fractures were seen to have lower median (IQR) ADL scores compared to non-hip fracture. These results were supported by a study conducted by Gill et al. (2013) which observed the relationship between serious fall injury and the outcomes after bone fractures among the older adult. The level of dependence on the basic activities of daily life such as bathing, dressing, walking and transferring among the older adult were consistently worse for hip fractures than for the other serious injuries [77]. Hip fractures were seen to have a great impact on patient life in terms of mobility and other aspects of life. Only 50 to 71% of patient who sustained a hip fracture are likely to regain their pre-fracture levels of mobility 12 months after the incident and 10 to 20% will be institutionalized permanently. Data from patients who have had a hip fracture showed that two years after a hip fracture, they were more likely to be community immobile and functionally dependent compared to controls [78,79]. A systematic study conducted to evaluate the outcomes of the patient who have had a hip fracture revealed that that the patient’s rate of recovery was very low. Apart from that, about 30% of hip fracture patients did not regain their pre-fracture level of independence in ADL [80]. Hip fractures were also considered as a type of bone fracture that contributes a huge impact on morbidity, mortality and cost of treatment among older adult [81].

The ability to walk before fracture is one of the factors that determine the success of returning to pre-fracture health function other than age, fracture types, comorbidities, functional status before fracture, anemia, subject placement status, and pain [63,82,83]. The present study showed that subjects using walking aids before fracture had significantly lower ADL scores compared to subjects that did not use walking aids before fracture. This was consistent with a study by Lin and Chang which concluded that the use of walking aids before fracture was one of the significant predictors for dependent on instrumental ADL [84]. These findings were inconsistent with a randomized controlled study by Karlsson et al. which observed the relation between walking ability and length of hospital stay after hip fracture. The study revealed no significant difference in the use of walking device at the 3- and 12-month follow up between the groups [85]. The inconsistent results on the use of walking aid before fracture required further studies.

There were improvements in the health outcomes of subjects aged more than 50 years old with lower limb fractures after 3 months of being discharged from the hospital. The care of geriatric patients with trauma should move beyond prevention of death and include multidisciplinary care targeted rehabilitation and prevention of permanent functional impairment. The older adult patients following trauma should be cared for by specialist nurses or caregiver. Telephone counselling for the fractured patients could be helpful.

This study has some limitations: first, it relied on self-report information, which could cause bias. The follow up period of patients at three months after discharge from hospital seems to be quite short. Ideally, the patients should be followed up at longer periods to have better reflections for activities of daily living (ADL) following trauma.

## 5. Conclusions

This was the first prospective study on the outcomes of older adult patients with lower limb fractures in Klang Valley, Malaysia. It has identified factors such as age, being female, having a hip fracture and using walking aid before fracture that may predict ADL following lower limb fracture. This information may provide inputs for treatment and rehabilitation programs for older patients with lower limb fracture.

## Figures and Tables

**Table 1:** The demographic data of the patients recruited in this study.

Demographic Profile	Ward Admission Phase *n* = 129 *N* (%)	Post Discharge Phase *n* = 89 *N* (%)
**Age (mean ± SD)**	71.29 ± 10.94	69.62 ± 10.68
**Age group**		
50–64	39 (30.2)	32 (36.0)
65–74	29 (22.5)	19 (21.3)
75–84	45 (34.9)	32 (36.0)
>84	16 (12.4)	6 (6.7)
**Ethnic/Race**		
Malay	48 (37.2)	33 (37.1)
Chinese	59 (45.7)	45 (50.6)
Indian	22 (17.1)	11 (12.4)
**Gender**		
Male	50 (38.8)	34 (38.2)
Female	79 (61.2)	55 (61.8)
**Anatomy of fracture parts**		
Hip and femur	84 (65.1)	51 (57.3)
Knees and calves	23 (17.8)	18 (20.2)
Ankle and foot	22 (17.1)	20 (22.5)
**Fracture type**		
Hip bone	78 (60.5)	46 (51.7)
Other than hip bone	51 (39.5)	43 (48.3)
**Ability to walk before fracture**		
Without walking aid	97 (75.2)	22 (24.7)
With walking aid	32 (24.8)	67 (75.3)

**Table 2:** Frequency and percentage of subjects for follow-up and the dropouts in the study.

Follow-Up Status	Frequency, *N*	Percentage (%)
Successful for follow-up visit	89	69.0
Died	14	10.9
Withdraw from the study	15	11.6
Uncontactable	11	8.5

**Table 3:** Frequency and percentage of subjects for all activities of daily living in all three phases.

ADL Scales	Pre-Fracture Phase *n* = 129 *N* (%)	Ward Admission Phase *n* = 129 *N* (%)	Post-Discharge Phase *n* = 89 *N* (%)
Bathing			
Independent	128 (99.2)	2 (1.6)	55 (61.8)
Dependent	1 (0.8)	127 (98.4)	34 (38.2)
Dressing			
Independent	128 (99.2)	14 (10.9)	57 (64.0)
Dependent	1 (0.8)	115 (89.1)	32 (36.0)
Transferring			
Independent	128 (99.2)	12 (9.3)	67 (75.3)
Dependent	1 (0.8)	117 (90.7)	22 (24.7)
Toileting			
Independent	128 (99.2)	3 (2.3)	55 (61.8)
Dependent	1 (0.8)	126 (97.7)	34 (38.2)
Continence			
Independent	113 (87.6)	22 (17.1)	66 (74.2)
Dependent	16 (12.4)	107 (82.9)	23 (25.8)
Feeding			
Independent	129 (100.0)	110 (85.3)	84 (94.4)
Dependent	0 (0.0)	19 (14.7)	5 (5.6)

**Table 4:** Logistic regression models for all ADL in all three phases. (**a**) Logistic regression for determinant factors for bathing activity, (**b**) dressing activity, (**c**) transferring activity (**d**) toileting activity, (**e**) continence activity (**f**) feeding activity.

(a) Logistic Regression for Bathing Activity			
Determinant Factors	Logistic Regression		
	95% CI	Odd Ratio (OR)	*p*-Value
**Age**	1.005 to 1.156	1.078	0.036
**Gender**			
Male	0.538 to 5.792	1.765	0.349
Female (R)			
**Type of fracture**			
Hip fracture	0.693 to 9.196	2.524	0.160
Non-hip fracture (R)			
**Ability to walk before the fracture**			
Walking aid			
Without walking aid (R)	1.688 to 20.383	5.866	0.005
**(b) Logistic Regression for Dressing Activity**			
**Determinant Factors**	**Logistic Regression**		
	**95% CI**	**Odd Ratio (OR)**	***p*-Value**
**Age**	1.037 to 1.227	11.233	0.001
**Gender**			
Male	0.360 to 4.950	1.335	0.665
Female (R)			
Type of fracture			
Hip fracture	0.464 to 8.607	1.999	0.352
Non-hip fracture (R)			
**Ability to walk before the fracture**			
Walking aid			
Without walking aid (R)	2.801 to 45.047	11.233	0.001
**(c) Logistic Regression for Transferring Activity**			
**Determinant Factors**	**Logistic Regression**		
	**95% CI**	**Odd Ratio (OR)**	***p*-Value**
**Age**	0.999 to 1.176	1.084	0.053
**Gender**			
Male	0.539 to 8.035	2.082	0.287
Female (R)			
**Type of fracture**			
Hip fracture			
Non-hip fracture (R)	0.558 to 13.089	2.702	0.217
**Ability to walk before the fracture**			
Walking aid			
Without walking aid (R)	1.147 to 11.843	3.684	0.029
**(d) Logistic Regression for Toileting Activity**			
**Determinant Factors**	**Logistic Regression**		
	**95% CI**	**Odd Ratio (OR)**	***p*-Value**
**Age**	1.022 to 1.149	1.073	0.043
**Gender**			
Male	0.506 to 5.123	1.610	0.420
Female (R)			
**Type of fracture**			
Hip fracture			
Non-hip fracture (R)	0.678 to 8.554	2.408	0.174
**Ability to walk before the fracture**			
Walking aid			
Without walking aid (R)	1.420 to 15.032	4.620	0.011
**(e) Logistic Regression for Continence Activity**			
**Determinant Factors**	**Logistic Regression**		
	**95% CI**	**Odd Ratio (OR)**	***p*-Value**
**Age**	1.024 to 1.220	1.118	0.013
**Gender**			
Male	1.050 to 18.046	4.354	0.043
Female (R)			
**Type of fracture**			
Hip fracture	0.249 to 4.876	1.102	1.102
Non-hip fracture (R)			
**Ability to walk before the fracture**			
Walking aid			
Without walking aid (R)	0.705 to 7.596	2.313	0.167
**(f) Logistic Regression for Feeding Activity**			
**Determinant Factors**	**Logistic Regression**		
	**95% CI**	**Odd Ratio (OR)**	***p*-Value**
**Age**	1.057 to 1.841	1.395	0.019
**Gender**			
Male			
Female (R)	0.044 to 9.734	0.657	0.760
**Type of fracture**			
Hip fracture			
Non-hip fracture (R)	0.013 to 9.863	0.363	0.548
**Ability to walk before the fracture**			
Walking aid			
Without walking aid (R)	0.840 to 199.860	12.955	0.067

R = reference variable.

**Table 5:** Minimum scores, maximum scores and mean scores for all three phases.

ADL Score	Pre-Fracture Phase *n* = 129	Ward Phase *n* = 129	Post-Discharge Phase *n* = 89
Minimum	3	0	0
Maximum	6	6	6
Mean	5.85	1.28	4.33

**Table 6:** Changes in ADL scores across all three phases.

Phases	ADL Score Median (IQR)	*p*-Value
Pre-fracture phase, *n* = 129	6 (0)	0.000
Ward phase, *n* = 129	1 (0)	
Post-discharge phase, *n* = 89	6 (4)	

**Table 7:** ADL scores and independent variables (age, gender, fracture type and ability to walk before fracture).

Independent Variables	Median (IQR)	*p*-Value
Age		
50–64	6 (0)	
>65	3 (5)	0.000
Gender		
Male	6(1)	
Female	6 (5)	0.021
Fracture type		
Hip fracture	3 (5)	
Non-hip fracture	6 (0)	0.000
Ability to walk before fracture		
Using walking aid	2 (3)	
Not using walking aid	6 (2)	0.000

## References

[1] Turło A., Cywińska A., Czopowicz M., Witkowski L., Niedźwiedź A., Słowikowska M., Borowicz H., Jaśkiewicz A., Winnicka A. (2015). The Effect of Different Types of Musculoskeletal Injuries on Blood Concentration of Serum Amyloid A in Thoroughbred Racehorses. PLoS ONE.

[2] Parmet S., Lynm C., Richard M. (2004). Bone Fractures. JAMA.

[3] Schuit S.C.E., van der Klift M., Weel A.E.A.M., de Laet C.E.D.H., Burger H., Seeman E., Hofman A., Uitterlinden A.G., van Leeuwen J.P.T.M., Polsa H.A.P. (2004). Fracture incidence and association with bone mineral density in elderly men and women: The Rotterdam Study. Bone.

[4] Harvey N., McCloskey E.V. (2016). Gaps and Solutions in Bone Health—A Global Framework for Improvement. World Osteoporosis Day. http://share.iofbonehealth.org/WOD/2016/thematic-report/WOD16-report-WEB-EN.pdf.

[5] Johnell O., Kanis J.A. (2006). An estimate of the worldwide prevalence and disability associated with osteoporotic fractures. Osteoporos. Int..

[6] Moayyeri A., Luben R.N., Bingham S.A., Welch A.A., Wareham N.J., Khaw K.T. (2008). Measured height loss predicts fractures in middle-aged and older men and women: The EPIC-Norfolk prospective population study. J. Bone Miner. Res..

[7] Clift B. (2008). Fractures of the Lower Limb (include Foot). https://www.gov.uk/government/uploads/system/uploads/attachment_data/file/384502/fractures_lower_limb.pdf.

[8] Kaye J.A., Jick H. (2004). Epidemiology of lower limb fractures in general practice in the United Kingdom. Inj. Prev..

[9] Jones G., Nguyen T., Sambrook P.N., Kelly P.J., Gilbert C., Eisman J.A. (1994). Symptomatic fracture incidence in elderly men and women: The Dubbo Osteoporosis Epidemiology Study (DOES). Osteoporos. Int..

[10] Melton L.J., Crowson C.S., O’Fallon W.M. (1999). Fracture incidence in Olmsted County, Minnesota: Comparison of urban with rural rates and changes in urban rates over time. Osteoporos. Int..

[11] Miller W. (1978). Survival and ambulation following hip fracture. J. Bone Jt. Surg. Am..

[12] Cummings S.R., Kelsey J.L., Nevitt M.C., O’Dowd K.J. (1985). Epidemiology of osteoporosis and osteoporotic fractures. Epidemiol. Rev..

[13] Jensen J.S., Bagger J. (1982). Long-term social prognosis after hip fractures. Acta Orthop. Scand..

[14] Cummings S.R., Melton L.J. (2002). Epidemiology and outcomes of osteoporotic fractures. Lancet.

[15] Colón-Emeric C.S., Saag K.G. (2006). Osteoporotic fractures in older adults. Best Pract. Res. Clin. Rheumatol..

[16] Haentjens P., Lamraski G., Boonen S. (2005). Costs and consequences of hip fracture occurrence in old age: An economic perspective. Disabil. Rehabil..

[17] Wiktorowicz M.E., Goeree R., Papaioannou A., Adachi J.D., Papadimitropoulos E. (2001). Economic implications of hip fracture: Health service use, institutional care and cost in Canada. Osteoporos. Int..

[18] Bouee S., Lafuma A., Fagnani F., Meunier P.J., Reginster J.Y. (2006). Estimation of direct unit costs associated with non-vertebral osteoporotic fractures in five European countries. Rheumatol. Int..

[19] Colon-Emeric C.S. (2012). Postoperative management of hip fractures: Interventions associated with improved outcomes. BoneKEy Rep..

[20] Safa A., Alavi N.M., Abedzadeh-Kalahroudi M. (2016). Predictive Factors of Dependency in Activities of Daily Living Following Limb Trauma in the Elderly. Trauma Mon..

[21] Brown C., Boling J., Manson M., Owens T., Zura R. (2012). Relation between prefracture characteristics and perioperative complications in the elderly adult patient with hip fracture. South. Med. J..

[22] Vochteloo A.J.H., Moerman S., Tuinebreijer W.E., Maier A.B., de Vries M.R., Bloem R.M., Nelissen R.G., Pilot P. (2013). More than half of hip fracture patients do not regain mobility in the first postoperative year. Geriatr. Gerontol. Int..

[23] Colon-Emeric C., Kuchibhatla M., Pieper C., Hawkes W., Fredman L., Magaziner J., Zimmerman S., Lyles K.W. (2003). The contribution of hip fracture to risk of subsequent fractures: Data from two longitudinal studies. Osteoporos. Int..

[24] Green S.B. (1991). How many subjects does it take to do a regression analysis?. Multivar. Behav. Res..

[25] Duca G.F.P., Da Silva M.C., Hallal P.C. (2009). Disability in relation to basic and instrumental activities of daily living among elderly subjects. Revista de Saúde Pública.

[26] Sharkawi M.A., Zulfarina S.M., Aqilah-SN S.M.Z., Isa N.M., Sabarul A.M., Nazrun A.S. (2016). Systematic Review on the Functional Status of Elderly Hip Fracture Patients using Katz Index of Activity of Daily Living (Katz ADL) Score. IMJM.

[27] Yu R., Wong M., Chang B., Lai X., Lum C.M., Auyeung T.W., Lee Y., Tsoi K., Lee R., Woo J. (2016). Trends in activities of daily living disability in a large sample of community-dwelling Chinese older adults in Hong Kong: An age-period-cohort analysis. BMJ Open.

[28] Al Aqqad S.H., Chen L.L., Shafie A.A., Hassali M.A., Tangiisuran B. (2014). The use of potentially inappropriate medications and changes in quality of life among older nursing home residents. Clin. Interv. Aging.

[29] Wallace M., Shelkey M. (2008). Katz Index of Independence in Activities of Daily Living (ADL). AJN.

[30] Garcia R., Leme M.D., Garcez-Leme L.E. (2006). Evolution of Brazilian Elderly with Hip Fracture Secondary to a Fall. CLINICS.

[31] Reijneveld S.A., Spijker J., Dijkshoorn H. (2007). Katz’ADL index assessed functional performance of Turkish, Moroccan, and Dutch elderly. J. Clin. Epidemiol..

[32] Katz S., Down T.D., Cash H.R., Grotza R.C. (1970). Progress in the Development of the Index of ADL. Gerontologist.

[33] Zweig M.H., Campbell G. (1993). Receiver-operating characteristic (ROC) plots: A fundamental evaluation tool in clinical medicine. Clin. Chem..

[34] Gold D.A. (2012). An examination of instrumental activities of daily living assessment in older adults and mild cognitive impairment. J. Clin. Exp. Neuropsychol..

[35] Wallace M., Shelkey M. (1999). Katz Index of Independence in Activities of Daily Living. J. Gerontol. Nurs..

[36] Wallace M., Shelkey M. (2012). Katz Index of Independence in Activities of Daily Living (ADL).

[37] Wiener J.M., Hanley R.J. (1989). Measuring the Activities of Daily Living among the Elderly: A Guide to National Surveys. https://aspe.hhs.gov/basic-report/measuring-activities-daily-living-among-elderly-guide-national-surveys.

[38] Puteh S.E.B.W., Bakar I.M.A., Borhanuddin B., Latiff K., Amin R.M., Sutan R. (2015). A Prevalence Study of the Activities of Daily Living (ADL) Dependency among the Elderly in Four Districts in Selangor, Malaysia. J. Epidemiol. Prev. Med..

[39] Tang Z., Wang H., Meng C., Wu X., Ericsson K., Winblad B., Pei J. (1999). The prevalence of functional disability in activities of daily living and instrumental activities of daily living among elderly Beijing Chinese. Arch. Gerontol. Geriatr..

[40] Matthews C.A., Whitehead W.E., Townsend M.K., Grodstein F. (2013). Risk Factors for Urinary, Fecal or Dual Incontinence in the Nurses’ Health Study. Obstet. Gynecol..

[41] Alavi N.M., Safa A., Abedzadeh-Kalahroudi M. (2014). Dependency in Activities of Daily Living Following Limb Trauma in Elderly Referred to Shahid Beheshti Hospital, Kashan-Iran in 2013. Arch. Trauma Res..

[42] Mossey J.M., Mutran E., Knott K., Craik R. (1989). Determinants of Recovery 12 months after hip fracture: The importance of Psychosocial factors. Am. J. Public Health.

[43] Kelsey J.L., Samelson E.J. (2009). Variation in Risk Factors for Fractures at Different Sites. Curr. Osteoporos. Rep..

[44] Curry L.C., Hogstel M.O., Davis G.C. (2003). Functional status in older women following hip fracture. J. Adv. Nurs..

[45] Svensson O., Stromberg L., Ohlen G., Lindgren U. (1996). Prediction of the outcome after hip fracture in elderly patients. J. Bone Jt. Surg. Br..

[46] Mehul R.S., Gina B.A., Philip W., Joseph D.Z., Kenneth J.K. (2001). Outcome after Hip fracture in individuals Ninety years of Age and Older. J. Orthop. Trauma.

[47] Wollinsky F.D., Fitzgerald J.F., Stump E.S. (1997). The Effect of Hip Fracture on Mortality, Hospitalization, and Functional Status: A Prospective Study. Am. J. Public Health.

[48] Alarcon T., Gonzalez-Montalvo J.I., Gotor P., Madero R., Otero A. (2011). Activities of daily living after hip fracture: Profile and rate of recovery during 2 years of follow-up. Osteoporos. Int..

[49] Carpenter G.I., Hastie C.L., Morris J.N., Fries B.E., Ankri J. (2006). Measuring change in activities of daily living in nursing home residents with moderate to severe cognitive impairment. BMC Geriatr..

[50] Magaziner J., Hawkes W., Hebel J.R., Zimmerman S.I., Fox K.M., Dolan M., Felsenthal G., Kenzora J. (2000). Recovery from hip fracture in eight areas of function. J. Gerontol. Med. Sci..

[51] Riemen A.H.K., Hutchison J.D. (2016). The multidisciplinary management of hip fractures in older patients. Orthop. Trauma.

[52] Karbakhshe M., Zargar M., Ershadi Z., Khaji A. (2006). Mechanism and outcome of hip fracture: A multi-center study. Tehran Univ. Med. J..

[53] Mobaleghi J., NotashAidin Y., Notash Ali Y., Ahmadi Amoli H., Borna L., NotashAnaram Y. (2014). Evaluation of trauma patterns and their related factors in Besat Hospital in Sanandaj in 2012. Sci. J. Kurdistan Univ. Med. Sci..

[54] Yu B., Chung M., Lee G., Lee J. (2014). Mortality and Morbidity in Severely Traumatized Elderly Patients. Korean J. Crit. Care Med..

[55] Kara H., Bayir A., Ak A., Akinci M., Tufekci N., Degirmenci S. (2014). Trauma in elderly patients evaluated in a hospital emergency department in Konya, Turkey: A retrospective study. Clin. Interv. Aging.

[56] Povoroznyuk V., Dedukh N., Makogonchuk A. (2014). Effect of aging on fracture healing. Gerontologija.

[57] Meyer M., Meyer R. (2006). Altered expression of mitochondrial genes in response to fracture in old rats. Acta Orthop..

[58] Nitti V.W. (2002). The Prevalence of Urinary Incontinence. Rev. Urol..

[59] Stenzelius K., Mattiasson A., Hallberg I.R., Westergren A. (2004). Symptoms of urinary and faecal incontinence among men and women 75+ in relations to health complaints and quality of life. Neurourol. Urodyn..

[60] Bertram M., Norman R., Kemp L., Vos T. (2011). Review of the long-term disability associated with hip fractures. Inj. Prev..

[61] Flikweert E.R., Izaks G.J., Reininga I.H., Wendt K.W., Stevens M. (2013). Evaluation of the effect of a comprehensive multidisciplinary care pathway for hip fractures: Design of a controlled study. BMC Musculoskelet. Disord..

[62] Norton R., Butler M., Robinson E., Lee-Joe T., Campbell A.J. (2000). Declines in physical functioning attributable to hip fracture among older people: A follow-up study of case–control participants. Disabil. Rehabil..

[63] Mariconda M., Costa G.G., Recano P., Orabona G., Gambacorta M., Misasi M. (2016). Factors Predicting Mobility and the Change in Activities of Daily Living After Hip Fracture: A 1-Year Prospective Cohort Study. J. Orthop. Trauma.

[64] Hu Y., Hu G., Hsu C., Hsieh S., Li C. (2012). Assessment of Individual Activities of Daily Living and its Association with Self-Rated Health in Elderly People of Taiwan. Int. J. Gerontol..

[65] Inagawa T., Hamagishi T., Takaso Y., Hitomi Y., Kambayashi Y., Hibino Y., Shibata A., Ngoc N.T.M., Okochi J., Hatta K. (2013). Decreased activity of daily living produced by the combination of Alzheimer’s disease and lower limb fracture in elderly requiring nursing care. Environ. Health Prev. Med..

[66] Heikkinen T., Jalovaara P. (2005). Four or Twelve Months Follow-Up in the Evaluation of functional Outcome After Hip fracture Surgery?. Scand. J. Surg..

[67] Takayama S., Iki M., Kusaka Y., Takagi H., Tamaki S. (2001). Factors that influence functional prognosis in elderly patients with hip fracture. Environ. Health Prev. Med..

[68] Koval K.J., Skovron M.L., Aharonoff G.B., Zuckerman J.D. (1998). Predictors of functional recovery after hip fracture in the elderly. Clin. Orthop. Relat. Res..

[69] Al-Ani A., Neander G., Samuelsson B., Blomfeldt R., Ekstrom W., Hedstrom M. (2013). Risk factors for osteoporosis are common in young and middle-aged patients with femoral neck fractures regardless of trauma mechanism. Acta Orthop..

[70] Alegre-López J., Cordero-Guevara J., Alonso-Valdivielso J.L., Fernández-Melón J. (2005). Factors associated with mortality and functional disability after hip fracture: An inception cohort study. Osteoporos. Int..

[71] Orive M., Aguirre U., Garcia-Gutierrez S., Las Hayas C., Bilbao A., Gonzalez N., Zabala J., Navarro G., Quintana J.M. (2015). Changes in health-related quality of life and activities of daily living after hip fracture because of a fall in elderly patients: A prospective cohort study. Int. J. Clin. Pract..

[72] Sylliaas H., Thingstad P., Wyller T.B., Helbostad J., Sletvold O., Bergland A. (2012). Prognostic factors for self-rated function and perceived health in patient living at home three months after a hip fracture. Disabil. Rehabil..

[73] Dudkiewicz I., Burg A., Salai M., Hershkovitz A.I. (2011). Gender differences among patients with proximal femur fractures during rehabilitation. Gend. Med..

[74] Samuelsson B., Hedström M.I., Ponzer S., Söderqvist A., Samnegård E., Thorngren K.G., Cederholm T., Sääf M., Dalen N. (2009). Gender differences and cognitive aspects on functional outcome after hip fracture—A 2 years’ follow-up of 2134 patients. Age Ageing.

[75] Solarino G., Vicenti G., Picca G., Rifino F., Carrozzo M., Moretti B. (2016). A review of gender differences in hip fracture anatomy, morbidity, mortality and function. Ital. J. Gend.-Specif. Med..

[76] Kempen G.I.J.M., Sanderman R., Scaf-Klomp W., Ormel J. (2003). Gender differences in recovery from injuries to the extremities in older persons. A prospective study. Disabil. Rehabil..

[77] Gill T.M., Murphy T.E., Gahbauer E.A., Allore H.G. (2013). The course of disability before and after a serious fall injury. JAMA Intern. Med..

[78] Vergara I., Vrotsou K., Orive M., Gonzalez N., Garcia S., Quintana J.M. (2014). Factors related to functional prognosis in elderly patients after accidental hip fractures: A prospective cohort study. BMC Geriatr..

[79] Ganezak M., Chrobrowski K., Korzen M. (2018). Predictors of a Change and Correlation in Activities of Daily Living after Hip Fracture in Elderly Patients in a Community Hospital in Poland: A Six-Month Prospective Cohort Study. Int. J. Environ. Res. Public Health.

[80] Dyer S.M., Crotty M., Fairhall N., Magaziner J., Beaupre L.A., Cameron I.D., Sherrington C. (2016). A critical review of the long-term disability outcomes following hip fracture. BMC Geriatr..

[81] Theander E., Jarnlo G., Ornstein E., Karlsson M.K. (2004). Activities of daily living similarly in hospital-treated patients with a hip fracture or a vertebral fracture: A one-year prospective study in 151 patients. Scand. J. Public Health.

[82] Kristensen M.T. (2011). Factors affecting functional prognosis of patients with hip fracture. Eur. J. Phys. Rehabil. Med..

[83] Montalban-Quesada S., Garcia-Garcia I., Moreno-Lorenzo C. (2012). Functional evolution in elderly individuals with hip fracture surgery. Revista da Escola de Enfermagem da USP.

[84] Lin P.C., Chang S.Y. (2004). Functional recovery among elderly people one year after hip fracture surgery. J. Nurs. Res..

[85] Karlsson A., Berggren M., Gustafson Y., Olofsson B., Lindelöf N., Stenvall M. (2016). Effects of Geriatric Interdisciplinary Home Rehabilitation on Walking Ability and Length of Hospital Stay After Hip Fracture: A Randomized Controlled Trial. JAMDA.

